# Effect estimate comparison between the prescription sequence symmetry analysis (PSSA) and parallel group study designs: A systematic review

**DOI:** 10.1371/journal.pone.0208389

**Published:** 2018-12-06

**Authors:** Demy L. Idema, Yuanyuan Wang, Michael Biehl, Peter L. Horvatovich, Eelko Hak

**Affiliations:** 1 Groningen Research Institute of Pharmacy, unit PharmacoTherapy, -Epidemiology & -Economics, University of Groningen, Groningen, The Netherlands; 2 Johann Bernoulli Institute for Mathematics and Computer Science, University of Groningen, Groningen, The Netherlands; 3 Groningen Research Institute of Pharmacy, unit Analytical Biochemistry, University of Groningen, Groningen, The Netherlands; Medizinische Universitat Wien, AUSTRIA

## Abstract

Prescription sequence symmetry analysis (PSSA), a case-only design introduced in 1996, has been increasingly used to identify unintentional drug effects, and has potential applications as a hypothesis-testing and a hypothesis-generating method, due to its easy application and effective control of time-invariant confounders. The aim of this study is to systematically compare effect estimates from the PSSA to effect estimates from conventional observational parallel group study designs, to assess the validity and constraints of the PSSA study design. We reviewed the MEDLINE, EMBASE, and Web of Science databases until February 2016 to identify studies that compared PSSA to a parallel group design. Data from the eligible articles was extracted and analyzed, including making a Bland-Altman plot and calculating the number of discrepancies between the designs. 63 comparisons (from two studies) were included in the review. There was a significant correlation (p < 0.001) between the effect estimates of the PSSA and the parallel group designs, but the bias indicated by the Bland-Altman plot (0.20) and the percentage of discrepancies (70–80%) showed that this correlation was not accompanied by a considerable similarity of the effect estimates. Overall, the effect estimates of the parallel group designs were higher than those of the PSSA, not necessarily further away from 1, and the parallel group designs also generated more significant signals. However, these results should be approached with caution, as the effect estimates were only retrieved from two separate studies. This review indicates that, even though PSSA has a lot of potential, the effect estimates generated by the PSSA are usually lower than the effect estimates generated by parallel group designs, and PSSA mostly has a lower power than the conventional study designs, but this is based on limited comparisons, and more comparisons are needed to make a proper conclusion.

## Introduction

Conventional observational parallel group studies, such as the cohort study and the case-control study, are still predominantly used to determine causal effects of risk factors and to assess drug safety [1]. An important limitation of these designs is that they use an exposed- and a reference group which are (frequently) not readily comparable. This can lead to biased results [2]. Case-only designs, such as the case-crossover study design and the self-controlled case-series are alternatives to parallel group designs, and they aim to decrease the possibility of introducing bias [3,4]. These designs are particularly useful to control for time-invariant confounders, even when these confounders are generally not recorded in the databases, such as genetic disposition, diet, and over-the-counter drug use [2,5].

**Fig 1:**
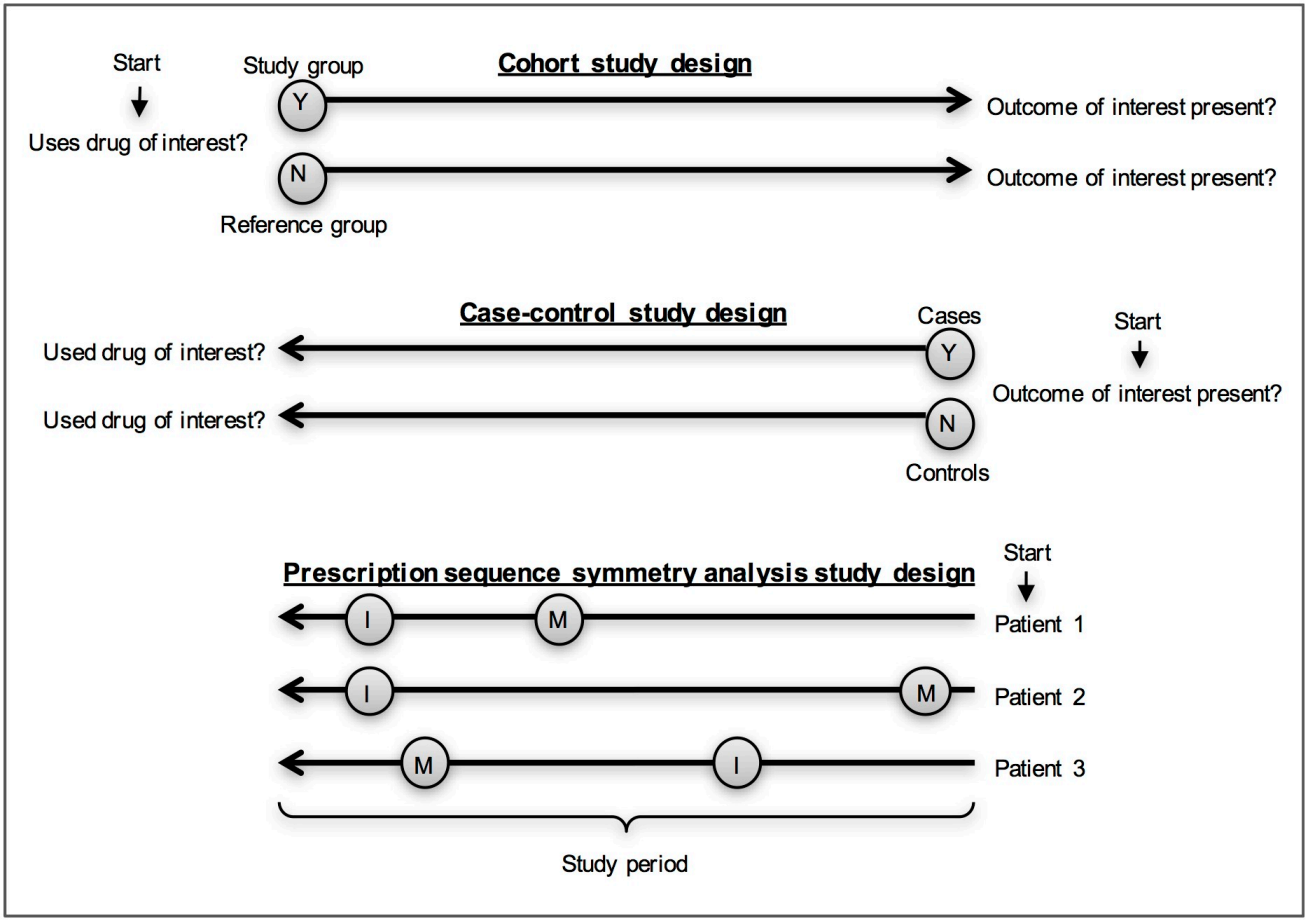
Schematic representation of the cohort-, case-control-, and PSSA study designs. Abbreviations: Y = yes, N = no, I = index drug, and M = marker drug.

In 1996, Hallas introduced another case-only study design: the prescription sequence symmetry analysis (PSSA) [6]. A key advantage of PSSA is that it can be used when there is an extensive amount of prescription data available, but no information is given for diagnoses, co-morbidities, and other possible confounders. In this study design, only patients who filled incident prescriptions for both the index drug (the drug under investigation) and the marker drug (the drug prescribed as a proxy/indicator for the outcome of interest, usually an unintentional effect of the index drug) during a predefined risk period are included in the analysis. The crude sequence ratio (SR) is calculated by dividing the number of patients who filled the prescription for the index drug first and the prescription for the marker drug second, by the number of patients with their prescriptions in the reverse order [7]. Since PSSA can be sensitive to temporal prescribing trends, the null-effect sequence ratio is also calculated. This is the expected SR in absence of a causal relationship between the index- and marker drug. A more detailed explanation of the originally proposed method to calculate the null-effect SR is given by Hallas [6]. In a study by Tsiropoulos *et al*. an adjustment to this calculation method is proposed, that takes into account risk periods that are shorter than the total study period [8]. By dividing the crude SR by the null-effect SR, the adjusted sequence ratio (ASR) is determined [9]. An ASR (including its confidence interval [CI]) above 1 indicates that the index drug may cause the adverse event for which the marker drug is prescribed, while an ASR (including its CI) below 1 suggests a possible protective effect [10]. A schematic representation of the cohort-, case-control, and PSSA study design is shown in Fig 1. Variations on the PSSA, such as (event) sequence symmetry analysis ((E)SSA), are also described in literature, and these variations also look at index- and marker events instead of drugs, such as surgeries or behavioral interventions [11].

PSSA has been used less frequently than other, more conventional, pharmacoepidemiologic study designs, and comparisons of PSSA to these designs are lacking [7–10, 12–26]. In this systematic review, we aim to compare PSSA to conventional study designs. In a previous study, the correlation between effect estimates from different designs has been measured, but correlation is not a measure of agreement between two effect measures [27,28]. Hence, information on the agreement and discrepancies between designs rather than the correlation between them is needed to assess the validity of the PSSA. We aim to systematically review articles that compared PSSA to a conventional study design to assess the effect of a medical intervention, to evaluate the differences between the study designs and assess possible limitations of the PSSA method. In this review, we will focus not only on the correlation between the effect estimates in PSSA and parallel designs, but especially on the agreement and discrepancies between them, and the direction of these discrepancies. Our results indicate that even though there is a strong correlation between the effect estimates from the two study designs, there is limited agreement between them and that there are systematic deviations.

## Methods

### Literature search strategies

The Preferred Reporting Items for Systematic Reviews and Meta-Analyses (PRISMA) checklist for this study can be found in the S1 File. We searched the MEDLINE and EMBASE databases from inception until February 2016 with the search terms “prescription event analysis” OR “symmetry principle” OR “prescription symmetry” OR “proximate clinical event ratio*” OR “sequence symmetry” OR “sequence-symmetry” OR “symmetry analys*” OR “sequence rat*” OR “prescription sequence”. We also performed a Web of Science cited reference search (also from inception until February 2016) for the article in which the PSSA method was introduced: “Hallas J”, “1996”, “Epidemiology”. All search results were limited to studies on humans, articles in English, and articles for which the abstract was available.

### Selection criteria

All identified articles were exported to RefWorks (ProQuest, Michigan). Title and abstract screening were performed and the full text of the relevant studies was reviewed for eligibility by two independent reviewers (D.L.I. and Y.W.). Disagreement between the reviewers was solved by consensus. Studies were eligible for inclusion in the review if they met the following criteria: the article compares (P)SSA to a conventional study design, the data for both study designs comes from the same data source and the definitions for the exposure(s) (index drug/event), outcome(s) (marker drug/event) and risk period(s) are equal for both study designs. Articles were excluded if they were systematic reviews, methodological studies, or studies with simulated data.

### Data extraction and analysis

For all articles that used (P)SSA as a study design identified by our search, whether the article was eligible for the review or not, the publication year was extracted to examine trends of application of this study design in time. These articles were split up into articles that used PSSA and articles that used another type of SSA, such as event sequence symmetry analysis. If both PSSA and SSA were used, the article was classified according to the principal study design, as identified by the article’s author.

From the eligible articles, we extracted the following data: author(s), year of publication, journal name, type of conventional study design and risk measure, exposure (index drug/event), outcome (marker drug/event), comparator used in the conventional study design, risk period(s), the conventional effect estimate and the PSSA effect estimate. If the study investigated multiple drug pairs, and there was not both a conventional effect estimate and a PSSA effect estimate for all of them, only the data for the drug pairs for which both effect estimates were reported was extracted.

As we compared multiple study designs to each other, rather than using different quality assessment tools for each study design, a method of quality assessment that we employed was to assess the reporting of potential confounders in the eligible articles. We based our assessment on the checklist by Pouwels *et al*., derived from the “Strengthening the Reporting of Observational Studies in Epidemiology” (STROBE) statement [29,30].

The effect estimates were exported to SPSS (IBM, New York, version 23), where they were analyzed using several approaches. First, a scatterplot was made of the effect estimates from the conventional study designs against the effect estimates from the PSSA study design to qualitatively examine potential differences in effect estimates. Second, the Spearman’s correlation coefficients were determined to evaluate the correlation between the effect estimates. Third, we examined whether the different study designs found the same significant associations. Fourth, because correlation may not be ideal to measure agreement between two types of study design, a Bland-Altman plot was made to assess this [28]. Moreover, the discrepancies between the effect estimates were evaluated, as previously done by Ioannidis et al: results were found to be discrepant if there was an absolute difference of 50% or more between the PSSA effect estimate and the parallel group design effect estimate on the natural logarithmic scale [31].

## Results

### Article identification

**Fig 2:**
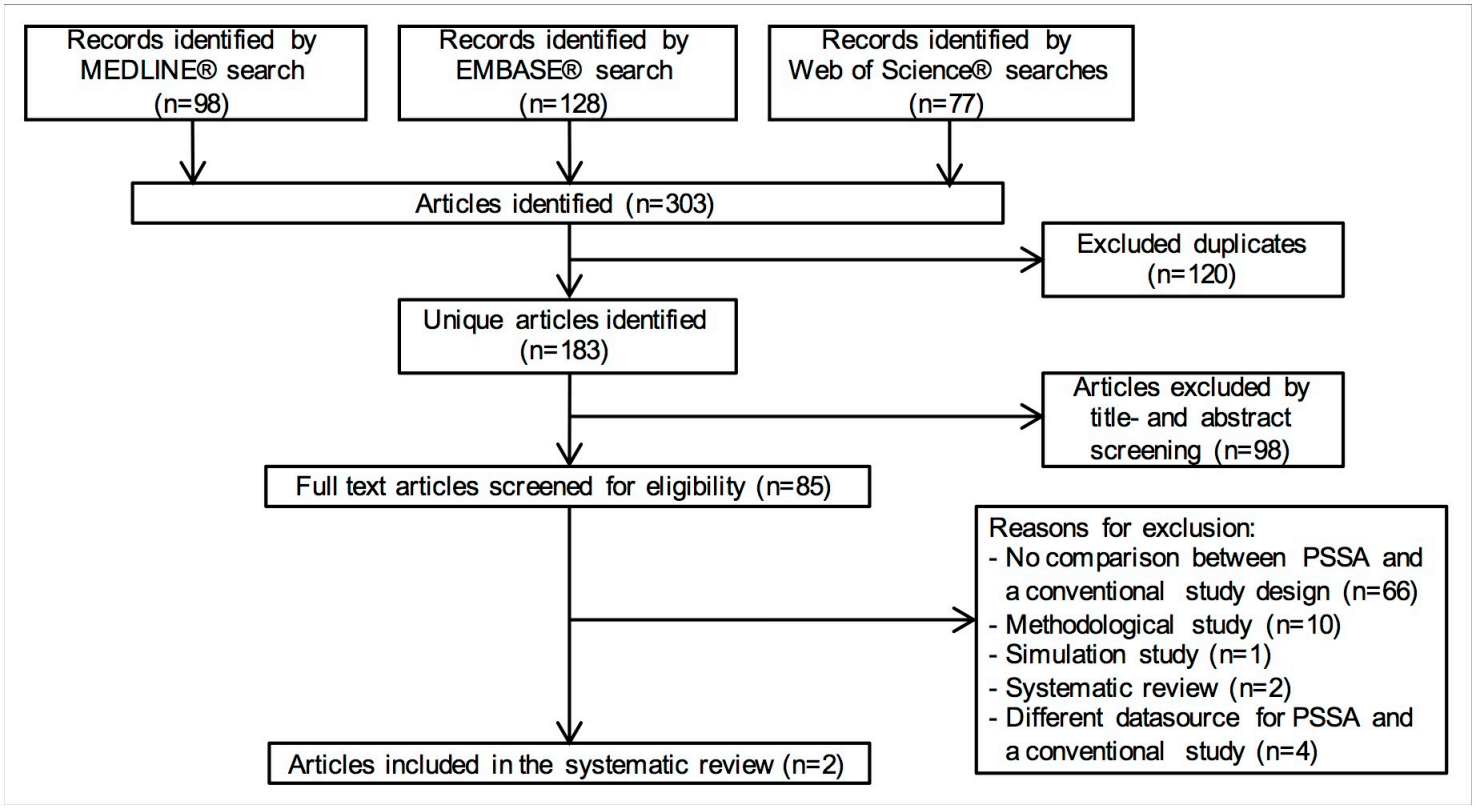
PRISMA flow diagram of the study selection process.

The search identified 183 unique articles. Based on the title and abstract screening, 85 potentially relevant articles were selected for full-text screening. After reviewing for eligibility, two articles were included into the review (Fig 2). The first article compared PSSA to both cohort- and nested case-control studies and the second article compared PSSA solely to a cohort study [27,32]. The data extracted from both articles is presented in Table 1.

**Table 1:** Main characteristics of articles included in this review.

Author, year	Journal	Number of drug pairs	Conventional design and effect estimate	Exposure	Comparator in the conventional design	Outcome	Risk period
Corrao, 2005 [27]	Pharmacoepidemiol. Drug Saf.	62	Cohort (SIR) and nested case-control (AOR)	Incident use of antibacterials	No incident use of antibacterials	Arrhythmia (incident anti-arrhythmic prescription)	18 months
Garrison, 2012 [32]	Arch. Intern. Med.	1	Cohort (HR)	Incident use of inhaled long-acting β_2_-agonists	Incident use of anticholinergics	Nocturnal muscle cramps (incident quinine prescription)	12 months

Abbreviations: SIR, standardized incidence ratio; AOR, adjusted odds ratio, HR, hazard ratio.

**Fig 3:**
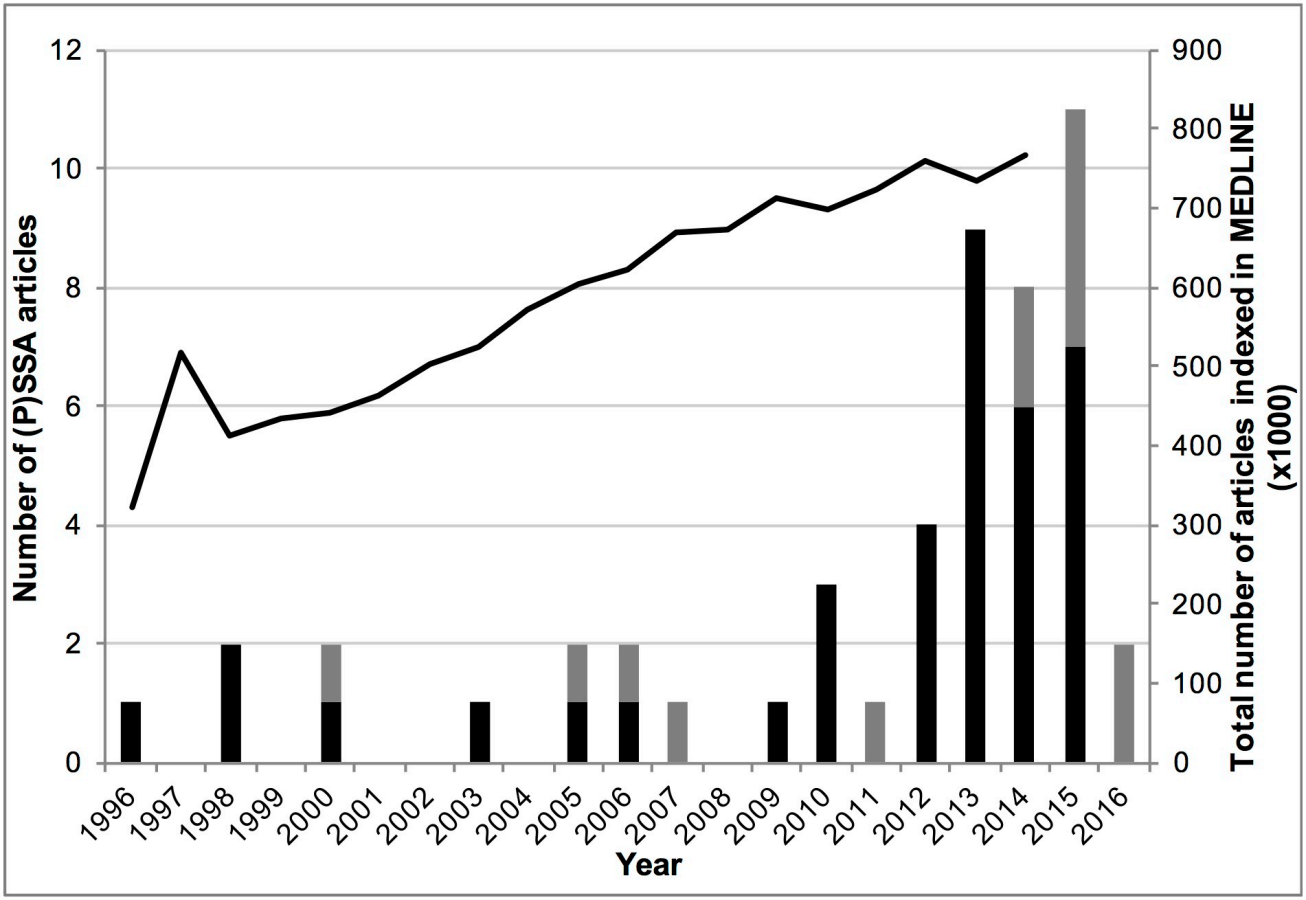
Number of (P)SSA articles published per year. Number of PSSA articles (black bar) and number of SSA articles (grey bar) published per year and the total number of articles indexed in MEDLINE (black line) per year after the introduction of the method in 1996 (first bar in the graph).

There were 50 articles (S2 File) that used (P)SSA to determine the effect of a medical intervention, and Fig 3 shows the number of these articles published per year. Even though the method was rarely used after its introduction in 1996, there is a clear increasing trend in the number of (published) PSSA studies during the last three to four years. For reference, the total number of articles indexed by MEDLINE per year has also been added to the figure [33]. The increase in the number of articles using the (P)SSA study design is relatively larger than the increase in total number of articles.

### Correlation analysis

A scatterplot was made of the conventional effect estimates against the PSSA effect estimates (S1 Fig). This scatterplot showed that there was a visible correlation between the effect estimates, but that for most of the investigated drug-pairs, the effect estimate from the conventional study designs was higher than the effect estimate from the PSSA study design, i.e. most of the data points were above the y = x reference line.

Spearman Rank-Order Correlation tests were performed (Table 2), first for all results taken into consideration, and followed by tests of the subsets of the results PSSA vs. cohort and PSSA vs. nested case-control designs. Their Spearman’s correlation coefficients were 0.621, 0.553, and 0.676, respectively. All results were highly statistically significant, with p ≤ 0.001.

**Table 2:** Summary of the Spearman-Rank Order Correlation analysis.

Dataset	N	Spearman’s correlation coefficient	*p*-value
All results	63	0.621	5.521·10^−8^
PSSA vs. cohort	35	0.553	0.001
PSSA vs. nested case-control	28	0.676	7.900·10^−5^

### Agreement and discrepancy analysis

Besides measuring correlation, a second approach used to compare the two methods was to assess if the different study designs found the same significant associations between the index- and marker drugs. In the first article, there were two significant signals found by all three methods, two signals that were only found with the conventional study designs and two additional signals that were identified only with the cohort design. In the second article, all designs found a statistically significant association for the investigated drug pair. So, combining data from both articles, the PSSA method identified less significant, potentially causal, associations between the index- and the marker drug than the two parallel group designs.

**Fig 4:**
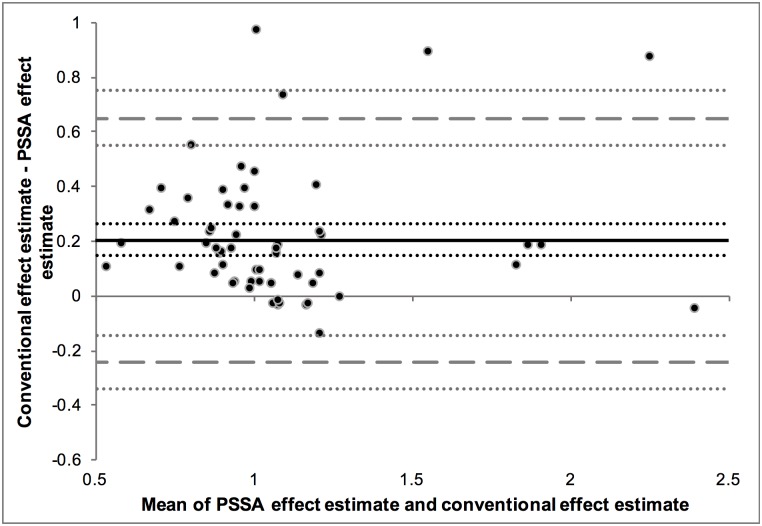
Bland-Altman plot of the difference between the effect estimates plotted against their mean. The black dots represent the difference against the mean of the effect estimate pairs; the black continuous line is the mean difference between the effect estimates (with 95% CI represented by the black dotted lines); the grey dashed lines represent the upper- and lower limit of agreement (with 95% CI’s represented by the grey dotted lines).

The third approach used to compare the effect estimates from the different study designs was to make a Bland-Altman plot to assess the degree of agreement between them (Fig 4). This figure shows that the mean difference between the conventional study design effect estimates and the PSSA study design effect estimates is 0.20 (95% CI [0.15, 0.26]), with the limits of agreement ranging from the lower limit of -0.24 (95% CI [-0.34, -0.14]) to the upper limit of 0.65 (95% CI [0.55, 0.75]). The Bland-Altman analysis shows that there is a degree of bias because the line of equality (the x-axis, y = 0) is not included in the confidence interval of the mean difference.

A fourth approach to compare the effect estimates is by determining discrepancies between them, as described before. The number and percentage of discrepancies are shown in Table 3. Even though the effect estimates were highly correlated, many results were characterized as discrepant when looking at the difference between them. Around 70–80% of all results were found to be discrepant, irrespective of whether it is a comparison to a cohort design or a comparison to a nested case-control design. Looking at these discrepancies, 92% of the cohort effect estimates and 96% of the nested case-control effect estimates were larger than the corresponding PSSA effect estimates. When assessing for the discrepancies whether the conventional effect estimate or the PSSA effect estimate was further away from 1, it was found that 33% of the cohort effect estimates and 36% of the nested case-control effect estimates were further away from 1 than the PSSA effect estimates.

**Table 3:** Number of discrepancies between PSSA and parallel group designs.

Dataset	Number (%) of discrepancies^a^	Number (%) of discrepancies for which the conventional effect estimate is larger^b^	Number (%) of discrepancies for which the conventional effect estimate is further away from 1^c^
All results (n = 63)	46 (73)	43 (94)	16 (35)
PSSA vs. cohort (n = 35)	24 (69)	22 (92)	8 (33)
PSSA vs. nested case-control (n = 28)	22 (79)	21 (96)	8 (36)

^a^Discrepancies were characterized by the natural logarithm of the PSSA effect estimate being ≥50% larger or smaller than the natural logarithm of the conventional study design effect estimate.

^b^The fraction of the total number of discrepancies for which the conventional effect estimate is larger than the PSSA effect estimate.

^c^The fraction of the total number of discrepancies for which the conventional effect estimate is further away from 1 than the PSSA effect estimate.

## Discussion

This study aimed to assess and quantify the correlation, agreement, and discrepancies between effect estimates from the PSSA and two conventional pharmacoepidemiologic study designs, the cohort- and nested case-control study design. We found that there was a significant correlation between the effect estimates of the PSSA and effect estimates of the conventional studies, but this strong correlation was not accompanied by similar effect estimates; there were systematic differences between the effect estimates generated by the two types of design. The Bland-Altman analysis showed significant bias between the effect estimates, with the effect estimates from the conventional study designs being, on average, 0.20 higher than the effect estimates from the PSSA.

The difference in effect size between the two types of design could originate from the use of a reference group in the conventional study designs, while the PSSA is a case-only design. Time-invariant confounders, whether registered or unregistered, such as advanced age, female gender, and hypochondriasis, may result in bias in parallel group designs (if they are not adjusted for) but not in the case-only PSSA [8]. Most comparisons (all apart from one) used in this review were derived from the study by Corrao *et al*. [27], and in this study, the comparisons from the cohort design were only adjusted for gender, age and month of observation, and the comparisons from the nested case-control design for gender, age, cumulative number of antibiotic prescriptions, and date of cohort entry. The PSSA method may inherently control for more confounders than this, and better confounder control could account for the difference in effect estimates.

However, this may not be the reason for the discrepancies if important assumptions for the validity of PSSA are not met. The assumptions of PSSA, based on the strengths and limitations of the method, are: there is an appropriate and specific indicator/proxy for the outcome, the proxy can be prescribed independently of the sequence of the exposure to the index drug and the occurrence of the outcome (e.g. if the outcome is fatal, the proxy could only be prescribed after incident index drug use), the outcome of interest has no effect on subsequent treatment, the effect of the exposure is transient, and the drug-induced symptom is relatively unknown to the prescribing physician [2,10,34–36]. Both articles discussed in this review mostly meet all assumptions, indicating that the differences between the effect estimates do not originate from invalid use of the PSSA design. The only possible problem is that the use of proxies for the outcomes may miss some cases, such as patients with unrecognized symptoms or patients who are hospitalized because of them, or it may include subjects taking the drug who do not have the outcome of interest.

The included articles used relatively long risk periods; 12- and 18 months. These are quite wide time-windows since exposure to the index drug shortly before the onset of the adverse event is more likely to be causal for the investigated exposures, especially for the antibacterials exposures from the study by Corrao *et al*. [27]. Here, more accurate effect estimates could be obtained if the risk window would be chosen more appropriately, i.e. would be shorter, based on the expected time that is needed for the manifestation of the adverse event. Also, to reduce the possibility of time-variant confounding, the risk period should be relatively small and should generally not exceed a couple of months to a year maximum. Note that for other drugs that are used for more extensive periods of time, and outcomes that may not be reported right away, longer risk periods may be appropriate.

Additionally, the underestimation of the effect size by the PSSA compared to the conventional study designs could be caused by the use of the relatively long risk periods in the PSSA. Using a longer risk period than necessary, especially in the case of the antibacterials exposure, could have diluted the signal by including more nonspecific sequences in the calculation of the adjusted sequence ratio. If the use of a longer risk period has more effect on the PSSA study design than on the other two designs, this could account (in part) for the lower effect estimates generated in this study design.

More differences were observed when assessing the number of adverse event signals (i.e. statistically significant results). There were cases where adverse event signals were only measured in the cohort- and nested case-control designs, but not in the PSSA. Since PSSA is only performed on subjects that filled incident prescriptions for both the index- and the marker drug, the sample size of the PSSA is smaller, and therefore the power lower, compared to that of the conventional designs. A possible solution for this was used by Pratt *et al*. in 2013, when they used a distributed network model to investigate the risk of acute hyperglycemia with antipsychotic use [17]. Using data from multiple countries increases the size of the population and the power of the analysis, and PSSA can therefore also detect rarer adverse events.

In general, for all arguments made above, it must be noted that our results are only based on two separate studies, and that it is therefore not possible to generalize these results to all studies that use PSSA and/or conventional study designs. A large part of our results (62 of the 63 comparisons included, 98%) are derived from a single study, and therefore these comparisons cannot be considered independent, and the results of our review are greatly dependent on the methodology and results used in that particular study. It may be the case that some part of the methodology of this study caused a systematic underestimation of the effect size of the PSSA study design compared to a conventional study design, and that application of the PSSA methodology is the cause of the discrepancy rather than the PSSA methodology itself. To be able to draw any generalizable conclusion from comparisons between the PSSA study design and conventional study designs, more independent comparisons between the two are greatly needed.

To assess the reporting of potential confounding in the articles used for this review, we used the checklist by Pouwels et al, derived from the “Strengthening the Reporting of Observational Studies in Epidemiology” (STROBE) statement [29,30]. In this checklist, there are eight items for the reporting of confounding. The first article in our review reported seven out of eight items from this checklist, while the second article reported six out of eight [27,32]. This is a high reporting quality compared to other articles, as demonstrated in the review by Pouwels et al, which found a median of four out of eight items reported [29].

One important advantage of PSSA is that it eliminates time-invariant confounders, but PSSA may still be sensitive to other types of confounding. Both articles calculated the adjusted sequence ratio to eliminate confounding by temporal prescribing trends and one article also adjusted for age and seasonal trends in drug prescription. Contra-indication was identified as a potential confounder, but this confounder is hard to control. A large part of the results of this review originates from a study that aimed to determine the effect of antibacterials on arrhythmia. The study split up the antibacterials into many subgroups and this division could have introduced confounding by contra-indication: physicians might only prescribe certain antibacterials to patients who are known to be at risk of arrhythmia. While this bias would not be the cause of the difference between the study designs, because this same division was made for the conventional study designs, it is an important confounder to take into consideration when using PSSA to compare subgroups of drugs prescribed for a similar indication. This is especially true when prescribing physicians are already aware of the potential adverse event, and may let this influence their prescribing behavior in choosing a specific drug for high-risk patients.

Also, when subjects consult their physician when they present with symptoms of an adverse event, some physicians may discontinue the index drug instead of prescribing the marker drug. In this case, these subjects would be missed by the PSSA, resulting in an underestimation of the effect estimate. Furthermore, it would also reduce the sample size, which further decreases the power of PSSA. However, most of the effect estimates from the conventional designs were also based on drug dispensing data, so this would not cause discrepancies between the effect estimates of both types of design studied in this review. Besides, it was demonstrated that even fairly well known adverse events are often treated by prescribing additional drugs, rather than discontinuing the drug that might have caused it [21].

### Strengths and limitations

This is the first systematic review that assesses the performance of PSSA compared to conventional study designs by reporting on the agreement and the discrepancies between the effect estimates and the direction of the discrepancies. A challenge that we have faced was the limited data available since empirical comparisons between PSSA and conventional designs are rare, and therefore it is hard to draw any definite conclusions from our results. We recognize that this could be due to the fact that researchers may perform both types of study and then publish the study with the most relevant results. This would underestimate the discrepancies between the study designs. Another possibility is that the results for both types of study were so similar that researchers only chose to publish the results of one of the two study designs. This would result in an overestimation of the difference between the study designs. Also, because the study designs use different effect measures, they may not be readily comparable, causing inconsistencies that are based on the different effect measures used rather than the different study designs. Therefore, there is a need for a quantitative statistical test that compares effect estimates from different risk measures.

## Conclusions

PSSA, due to its simple and quick implementation and its ability to eliminate time-invariant confounding has a lot of potential in assessing drug safety. However, our results indicate that PSSA lacks power in many situations, and its results often significantly deviate from effect estimates generated by conventional parallel group study designs. PSSA might, therefore, be more suitable as a hypothesis-generating design, that should be followed by a more conventional parallel group design for hypothesis-testing and confirmation. Our results should be approached with some caution, though, as they are only based on two independent studies. To get a better understanding of the practical differences between the two types of designs, and to be able to make any generalizations, more comparisons between PSSA and parallel group designs are required. Future studies should also compare PSSA to randomized controlled trials, to assess how PSSA performs against the study design considered to be the golden standard.

## Supporting information

S1 File PRISMA checklist. (PDF)

S2 File References for all (P)SSA articles identified in the literature search. All articles that addressed (P)SSA as a study design are included in this list, regardless of whether they were eligible to be included in the systematic review.
(PDF)

S1 Fig Scatterplot of the conventional effect estimates versus the PSSA effect estimates. Grey diamonds (with continuous grey line): PSSA vs. cohort with accompanying trend line; black squares (with continuous black line): PSSA vs. nested case-control with accompanying trend line. The dashed grey line represents the line y = x. Abbreviations: SIR, standardized incidence ratio; HR, hazard ratio; AOR, adjusted odds ratio; ASR, adjusted sequence ratio.
(PDF)
