# Supplementary material for: Effect estimate comparison between the prescription sequence symmetry analysis (PSSA) and parallel group study designs: A systematic review
Source: PLoS One. 2018 Dec 6;13(12):e0208389. doi: 10.1371/journal.pone.0208389 (PMC6283622; doi:10.1371/journal.pone.0208389)
Supplement: S2 File — All articles that addressed (P)SSA as a study design are included in this list, regardless of whether they were eligible to be included in the systematic review. (PDF) [file pone.0208389.s002.pdf]

References for all (P)SSA articles identified in the literature search.

1. Almqvist C, Wettermark B, Hedlin G, Ye W, Lundholm C. Antibiotics and asthma medication in a large register-based cohort study – confounding, cause and effect. Clin Exp Allergy. 2012;42: 104-111. doi:10.1111/j.1365-2222.2011.03850.x

2. Amundsen MO, Engdahl B, Berg C, Nordeng H. Use of psychotropic drugs and analgesics among users of antiobesity drugs – a population based study. Pharmacoepidemiol Drug Saf. 2010;19: 273-279. doi:10.1002/pds.1896

3. Baine WB, Kazakova SV. An analysis of administrative data found that proximate clinical event ratios provided a systematic approach to identifying possible iatrogenic risk factors or complications. J Clin Epidemiol. 2005;58: 162-170.

4. Bytzer P, Hallas J. Drug-induced symptoms of functional dyspepsia and nausea. A symmetry analysis of one million prescriptions. Aliment Pharmacol Ther. 2000;14: 1479-1484.

5. Caughey GE, Roughead EE, Pratt N, Shakib S, Vitry AI, Gilbert AL. Increased risk of hip fracture in the elderly associated with prochlorperazine: is a prescribing cascade contributing? Pharmacoepidemiol Drug Saf. 2010;19: 977-982. doi:10.1002/pds.2009

6. Caughey GE, Roughead EE, Pratt N, Killer G, Gilbert AL. Stroke risk and NSAIDs: an Australian population-based study. Med J Aust. 2011;195: 525-529.

7. Chen T, Chen L, Knaggs RD. Prevalence of antidepressants prescribed to tramadol users in the UK primary care setting – a prescription sequence symmetry analysis. Value Health. 2015;18: A661. doi:10.1016/j.jval.2015.09.2399

8. Cher DJ. Myocardial infarction and acute cholecystitis: an application of sequence symmetry analysis. Epidemiology. 2000;11: 446-449.

9. Cole JA, Farraye FA, Cabral HJ, Zhang Y, Rothman KJ. Irritable bowel syndrome and hysterectomy: a sequence symmetry analysis. Epidemiology. 2007;18(6):837-838.

10. Corrao G, Botteri E, Bagnardi V, Zambon A, Carobbio A, Falcone C, et al. Generating signals of drug-adverse effects from prescription databases and application to the risk of arrhythmia associated with antibacterials. Pharmacoepidemiol Drug Saf. 2005;14: 31-40.

11. Fujimoto M, Higuchi T, Hosomi K, Takada M. Association of statin use with storage lower urinary tract symptoms: data mining of claims database. J Pharmacovigilance. 2014;2: 147. doi:10.4172/2329-6887.1000147

12. Fujimoto M, Higuchi T, Hosomi K, Takada M. Association between statin use and cancer: data mining of a spontaneous reporting database and a claims database. Int J Med Sci. 2015; 12: 223-233. doi:10.7150/ijms.10656

13. Fujimoto M, Higuchi T, Hosomi K, Takada M. Association between renin-angiotensin system inhibitors and cancer risk; data mining of a spontaneous reporting database and a claims database. Pharmacoepidemiol Drug Saf. 2015;24: 474. doi:10.1002/pds

14. Garrison SR, Dormuth CR, Morrow RL, Carney GA, Khan KM. Nocturnal leg cramps and prescription use that precedes them: a sequence symmetry analysis. Arch Intern Med. 2012;172: 120-126. doi:10.1001/archinternmed.2011

15. Hachiken H, Murai A, Wada K, Kuwahara T, Hosomi K, Takada M. Difference between the frequencies of antisecretory drug prescriptions in users of buffered vs. enteric-coated low-dose aspirin therapies. Int J Clin Pharmacol Ther. 2013;51: 807-815. doi:10.5414/CP201914

16. Hallas J. Evidence of depression provoked by cardiovascular medication: a prescription sequence symmetry analysis. Epidemiology. 1996;7: 478-484.

17. Hallas J, Bytzer P. Screening for drug-related dyspepsia: an analysis of prescription symmetry. Eur J Gastroenterol Hepatol. 1998;10: 27-32.

18. Hashimoto M, Hashimoto K, Ando F, Kimura Y, Nagase K, Arai K. Prescription rate of medications potentially contributing to lower urinary tract symptoms and detection of adverse reactions by prescription sequence symmetry analysis. J Pharm Health Care Sci. 2015;1: 7. doi:10.1186/s40780-014-0004-1

19. Hersom K, Neary MP, Levaux HP, Klaskala W, Strauss JS. Isotretinoin and antidepressant pharmacotherapy: a prescription sequence symmetry analysis. J Am Acad Dermatol. 2003;49: 424-432.

20. Hosomi K. Association of antipsychotic use with extrapyramidal symptoms: data mining of the Japanese national receipt database. Pharmacoepidemiol Drug Saf. 2015;24: 327. doi:10.1002/pds

21. Ishiguro C, Kajiyama K, Uyama Y, Kondo E. Risk evaluation of drug-induced parkinsonism associated with antipsychotics based on two different study designs. Pharmacoepidemiol Drug Saf. 2015;24: 328. doi:10.1002/pds

22. Kalisch Ellett LM, Pratt NL, Barratt JD, Roughead EE. Urinary incontinence: a poorly recognized adverse effect of medicines. Pharmacoepidemiol Drug Saf. 2013;22: 475. doi:10.1002/pds.3512

23. Kalisch Ellett LM, Pratt NL, Barratt JD, Rowett D, Roughead EE. Risk of medication-associated initiation of oxybutynin in elderly men and women. J Am Geriatr Soc. 2014;62: 690-695. doi:10.1111/jgs.12741

24. Lai EC, Yang YH, Lin SJ, Hsieh CY. Use of antiepileptic drugs and risk of hypothyroidism. Pharmacoepidemiol Drug Saf. 2013;22: 1071-1079. doi:10.1002/pds.3498

25. Lai EC, Pottegård A, Hallas J, Yang YH. Use of antiepileptic drugs and risk of infection in Taiwan and Denmark: a collaborative cross-national sequence symmetry analysis. Pharmacoepidemiol Drug Saf. 2013;22: 94. doi:10.1002/pds.3512

26. Lai EC, Hsieh CY, Kao Yang YH, Lin SJ. Detecting potential adverse reactions of sulpride in schizophrenic patients by prescription sequence symmetry analysis. PLoS One. 2014;9: e89795. doi:10.1371/journal.pone.0089795

27. Lindberg G, Hallas J. Cholesterol-lowering drugs and antidepressants – a study of prescription symmetry. Pharmacoepidemiol Drug Saf. 1998;7: 399-402.

28. Pouwels KB, Visser ST, Bos HJ, Hak E. Angiotensin-converting enzyme inhibitor treatment and the development of urinary tract infections: a prescription sequence symmetry analysis. Drug Saf. 2013;36: 1079-1086. doi:10.1007/s40264-013-0085-z

29. Pouwels KB, Bos JH, Hak E. ACE inhibitors and urinary tract infections. Epidemiology. 2014;25: 466-467. doi:10.1097/EDE.0000000000000088

30. Pouwels KB, Kalkman GA, Schagen D, Visser ST, Hak E. Is combined use of SSRIs and NSAIDs associated with an increased risk of starting peptic ulcer treatment? Br J Clin Pharmacol. 2014;78: 192-193. doi:10.1111/bcp.12300

31. Pratt N, Andersen M, Bergman U, Choi NK, Gerhard T, Huang C, et al. Multi-country rapid adverse drug event assessment: the Asian Pharmacoepidemiology Network (AsPEN) antipsychotic and acute hyperglycaemia study. Pharmacoepidemiol Drug Saf. 2013;22: 915-924. doi:10.1002/pds.3440

32. Pratt N, Chan EW, Choi NK, Kimura M, Kimura T, Kubota K, et al. Prescription sequence symmetry analysis: assessing risk, temporality, and consistency for adverse drug reactions

across datasets in five countries. Pharmacoepidemiol Drug Saf. 2015;24: 858-864. doi:10.1002/pds.3780

33. Rasmussen L, Hallas J, Madsen KG, Pottegård A. Cardiovascular drugs and erectile dysfunction - a symmetry analysis. Br J Clin Pharmacol. 2015;80: 1219-1223.

34. Roughead EE, Kalisch LM, Pratt NL, Killer G, Barnard A, Gilbert AL. Managing glaucoma in those with co-morbidity: not as easy as it seems. Ophthalmic Epidemiol. 2012;19: 74-82. doi:10.3109/09286586.2011.638743

35. Roughead EE, Chan EW, Choi NK, Kimura M, Kimura T, Kubota K, et al. Variation in association between thiazolidinediones and heart failure across ethnic groups: retrospective analysis of large healthcare claims databases in six countries. Drug Saf. 2015;38: 823-831. doi:10.1007/s40264-015-0318-4

36. Silwer L, Petzold M, Hallas J, Lundborg CS. Statins and nonsteroidal anti-inflammatory drugs – an analysis of prescription symmetry. Pharmacoepidemiol Drug Saf. 2006;15: 510-511.

37. Takada M, Fujimoto M, Yamazaki K, Takamoto M, Hosomi K. Association of statin use with sleep disturbances: data mining of a spontaneous reporting database and a prescription database. Drug Saf. 2014;37: 421-431. doi:10.1007/s40264-014-0163-x

38. Takada M, Fujimoto M, Hosomi K. Difference in risk of gastrointestinal complications between users of enteric-coated and buffered low-dose aspirin. Int J Clin Pharmacol Ther. 2014;52: 181-191. doi:10.5414/CP201997

39. Takada M, Fujimoto M, Kanou M, Hosomi K. Inverse association between antiepileptic drugs and cancers; data mining of large medical databases. Pharmacoepidemiol Drug Saf. 2015;24: 451-452. doi:10.1002/pds

40. Takada M, Fujimoto M, Motomura H, Hosomi K. Inverse association between sodium channel-blocking antiepileptic drug use and cancer: data mining of spontaneous reporting and claims databases. Int J Med Sci. 2016;13: 48-59. doi:10.7150/ijms.13834

41. Takeuchi Y, Kajiyama K, Ishiguro C, Uyama Y. Atypical antipsychotics and the risk of hyperlipidemia: a sequence symmetry analysis. Drug Saf. 2015;38: 641-650. doi: 10.1007/s40264-015-0298-4

42. Thacker EL, Schneeweiss S. Initiation of acetylcholinesterase inhibitors and complications of chronic airways disorders in elderly patients. Drug Saf. 2006;29: 1077-1085.

43. Tsiropoulos I, Andersen M, Hallas J. Adverse events with use of antiepileptic drugs: a prescription and event symmetry analysis. Pharmacoepidemiol Drug Saf. 2009;18: 483-491. doi:10.1002/pds.1736

44. Van Boven JF, De Jong-van den Berg LT, Vegter S. Inhaled corticosteroids and the occurrence of oral candidiasis: a prescription sequence symmetry analysis. Drug Saf. 2013;36: 231-236. doi:10.1007/s40264-013-0029-7.

45. Vegter S, De Jong-van den Berg LT. Misdiagnosis and mistreatment of a common side-effect – angiotensin-converting enzyme inhibitor-induced cough. Br J Clin Pharmacol. 2010;69: 200-203. doi:10.1111/j.1365-2125.2009.03571.x

46. Vegter S, De Boer P, Van Dijk KW, Visser S, De Jong-van den Berg LTW. Misdiagnosis of ACE-inhibitor-induced cough occurs frequently and decreases therapy compliance. PW Wetenschappelijk Platform. 2012;6: a1228.

47. Vegter S, De Boer P, Van Dijk KW, Visser S, De Jong-van den Berg LT. The effects of antitussive treatment of ACE inhibitor-induced cough on therapy compliance: a prescription sequence symmetry analysis. Drug Saf. 2013;36: 435-439. doi:10.1007/s40264-013-0024-z

48. Wahab IA, Pratt NL, Kalisch LM, Roughead EE. Comparing time to adverse drug reaction signals in a spontaneous database and a claims database: a case study of rofecoxib-induced myocardial infarction and rosiglitazone-induced heart failure signals in Australia. Drug Saf. 2014;37: 53-64. doi:10.1007/s40264-013-0124-9.

49. Wahab IA, Pratt NL, Ellet LK, Roughead EE. Sequence symmetry analysis as a signal detection tool for potential heart failure adverse events in an administrative claims database. Drug Saf. 2016;39: 347-354. doi:10.1007/s40264-015-0391-8

50. Wang TT, Pratt N, Killer G, Roughead EE, Griffiths J. Proton pump inhibitors and clostridium difficile-associated disease in Canadian and Australian community settings – a prescription sequence symmetry analysis. Pharmacoepidemiol Drug Saf. 2013;22: 187-188. doi:10.1002/pds.3512
