# Supplementary figures and images for: Effect estimate comparison between the prescription sequence symmetry analysis (PSSA) and parallel group study designs: A systematic review
Source: PLoS One. 2018 Dec 6;13(12):e0208389. doi: 10.1371/journal.pone.0208389 (PMC6283622; doi:10.1371/journal.pone.0208389)

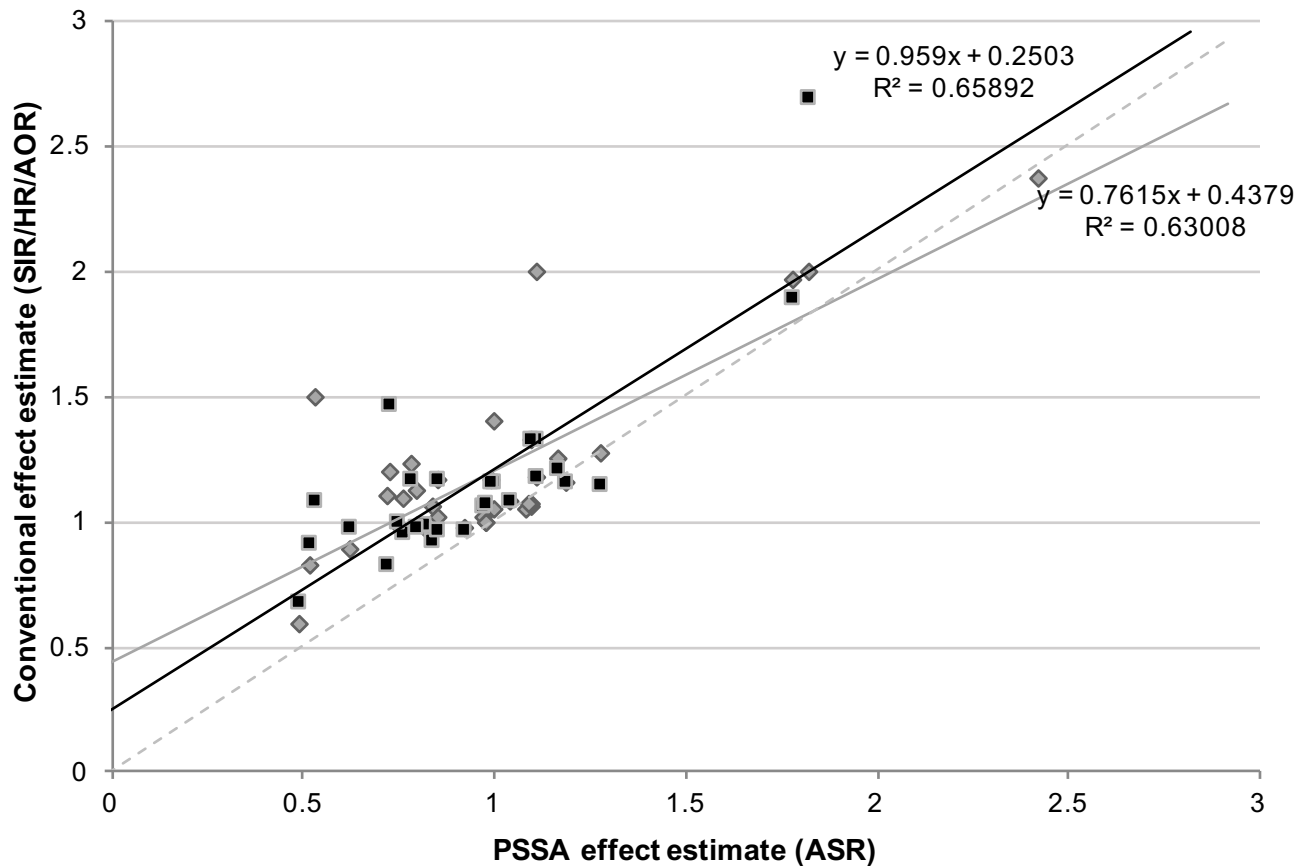

Supplement: S1 Fig — Grey diamonds (with continuous grey line): PSSA vs. cohort with accompanying trend line; black squares (with continuous black line): PSSA vs. nested case-control with accompanying trend line. The dashed grey line represents the line y = x. Abbreviations: SIR, standardized incidence ratio; HR, hazard ratio; AOR, adjusted odds ratio; ASR, adjusted sequence ratio. (PDF) [file pone.0208389.s003.pdf]
